# The hypertension and hyperlipidemia status among type 2 diabetic patients in the community and influencing factors analysis of glycemic control

**DOI:** 10.1186/s13098-023-01013-0

**Published:** 2023-04-13

**Authors:** Zhiyu Zhai, Yunou Yang, Guozhen Lin, Weiquan Lin, Jiagang Wu, Xiangyi Liu, Shijia Zhang, Qin Zhou, Hui Liu, Guang Hao

**Affiliations:** 1 Department of Non-Communicable Chronic Diseases Control and Prevention, Guangzhou Center for Disease Control and Prevention, No. 1 Qide Road, Guangzhou, 510440 Guangdong China; 2 Department of Public Health and Preventive Medicine, School of Medicine, Jinan University, 601 West Huangpu Road, Guangzhou, 510632 Guangdong China; 3 Guangdong Key Laboratory of Environmental Exposure and Health, Jinan University, Guangzhou, China

**Keywords:** Diabetes, Hypertension, Hyperlipidemia, Blood glucose control

## Abstract

**Objective:**

To understand the prevalence of hypertension and hyperlipidaemia as well as the current status of glycaemic control and its influencing factors among type 2 diabetes mellitus patients in the community in South China, and to provide recommendations for the prevention and control of diabetes.

**Methods:**

Questionnaires, physical examinations and laboratory tests were conducted on patients with type 2 diabetes mellitus who participated in the National Basic Public Health Service Programme in Guangzhou in 2020. The chi-square test, t-test and multi-factor unconditional logistic regression analysis were performed using R 4.1.2 software.

**Result:**

Among 127,423 type 2 diabetic patients in Guangzhou, 57,695 achieved glycemic control standards, with a glycemic control rate of 45.28%.In this study, the proportion of T2DM patients with hypertension and hyperlipidaemia together was 27.79%, The percentage of T2DM patients with hypertension alone and hyperlipidaemia alone was 28.34% and 20.53% respectively, and the rate of no complications was 23.34%. There was a statistically significant difference in the rate of glycaemic control between the different disease combination states (*P* < 0.05). The glycaemic control rate was 47.67% in diabetic patients without hypertension and hyperlipidaemia, 52.54% and 37.24% in those with combined hypertension alone and hyperlipidaemia alone respectively, compared to 41.80% in diabetic patients with hypertension and hyperlipidaemia. After adjusting for all covariates, multivariate analysis showed that combined hypertension alone was associated with good glycaemic control (OR 0.817, 95% CI 0.791, 0.843, *P* < 0.001),when using comorbid T2DM as a control group, combined hyperlipidaemia alone, combined hypertension and hyperlipidaemia were associated with poor glycaemic control (OR 1.521, 95% CI 1.470,1.574, *P* < 0.001 and OR 1.250, 95% CI 1.211,1.291, *P* < 0.001), Subgroup analyses as well as multifactorial unconditional logistic regression analyses showed that patients with type 2 diabetes who were overweight and obese, smoked, drank alcohol, had a diagnosis of diabetes for ≥ 6 years, had fair or poor adherence and had a family history of diabetes had lower rates of glycaemic control.

**Conclusion:**

The results of this study showed that the co-morbidity of hypertension and hyperlipidaemia was high and prevalent among diabetic patients in Guangzhou. Moreover, glycaemic control of T2DM patients with hyperlipidaemia was lower than other diabetic patients. Obesity and overweight, poor lifestyle and dietary habits are also major factors affecting the treatment and control of T2D patients in this region. Therefore, comprehensive measures should be actively taken to control blood glucose levels in type 2 diabetic patients by also incorporating lipid management into the community and strictly controlling lipid levels.

**Supplementary Information:**

The online version contains supplementary material available at 10.1186/s13098-023-01013-0.

## Introduction

The type 2 diabetes mellitus (T2DM) is a group of metabolic diseases characterised by hyperglycaemia due to defects in insulin secretion, insulin action or both [1].With socio-economic development, T2DM has become an important public health issue affecting the health of the Chinese population. According to the International Diabetes Federation and the Global Burden of Disease (GBD) [2], approximately 462 million people have been diagnosed with diabetes, equivalent to 6.28% of the world's population, and nearly 4 million people worldwide die of hyperglycaemia and the complications each year, with approximately US$850 billion spent on healthcare for adults with diabetes in 2017 [3]. Epidemiological findings showed that the prevalence of diabetes in China ranges from 9.7 to 11.6%, and the situation has not improved significantly in recent years, so the overall prevention and control of diabetes in China still faces a daunting challenge [4]. The World Health Organization (WHO) and other authorities believe that only active prevention and intervention can slow down and gradually stop the onset and progression of diabetes at its root [5].

Studies have shown that comorbidities such as cardiovascular disease (CVD), hypertension (HTN), dyslipidaemia and renal insufficiency often coincide with a diagnosis of T2D [6–8]. Therefore, a focus on T2DM needs to be accompanied by a focus on its comorbidities. For example, 60% of people with hypertension also have diabetes and 73% have dyslipidaemia [9]. Among people with diabetes, 30% may also have dyslipidaemia [10]. Previous studies have shown that the presence of multiple risk factors additionally increases the risk of cardiovascular disease [11]. Coexisting comorbidities, particularly the presence of any form of combination of diabetes, hypertension and dyslipidaemia have an even higher risk of developing cardiovascular and renal disease than each of these conditions alone [12].

Evidence suggested that the primary treatment goal for all patients with diabetes was to maintain good glycaemic control to prevent organ damage and microvascular and macrovascular complications. However, most patients failed to achieve good glycaemic control and the causes of poor glycaemic control are complex and multifactorial [13, 14]. Patients with poor glycaemic control may experience cognitive impairment, immune dysfunction, as well as hospital admissions and diabetic complications [15].

Guangzhou is a representative of a developed city in China. It has a developed economy and abundant medical resources, but it is also accompanied by a serious ageing population and a rising incidence of chronic diseases. In order to understand the current situation of hypertension and hyperlipidaemia complications as well as glycaemic control among diabetic patients in Guangzhou and their related influencing factors. This study was conducted on patients with type 2 diabetes managed by the National Basic Public Health Service in Guangzhou in 2020 to provide a reference basis for developing more targeted diabetes prevention and control measures.

## Research design and methods

### Data sources and study population

The study took patients with type 2 diabetes mellitus who were included in the management of national basic public health services in Guangzhou in 2020 as the study subjects, and according to the inclusion criteria, the included study subjects must have complete demographic and physical examination data, so patients with missing demographic statistics and those who did not participate in comprehensive health examination (i.e., missing physical examination data) in the current year were excluded, and before collecting questionnaires in different areas of Guangzhou, they must Comply with the following quality control guidelines: (1) Develop a manual for investigators, strictly train investigators, unify survey methods, improve survey quality, and only participate in formal surveys after passing the assessment; (2) Physical measurements, body composition determination, blood sample collection and laboratory testing tests are conducted using national standard methods, unified methods and reagents, and standardized operations; (3) All investigators count questionnaires immediately after their collection quantity, and the quality of questionnaires was strictly controlled; (4) the questionnaires were entered using a two-person parallel entry method and were tested for consistency. A total of 127,423 type 2 diabetic patients with complete medical examination data were included in this study.

### Research method

Gender, age, education, smoking status, alcohol consumption, physical activity, history of hypertension, hyperlipidemia, family history of diabetes and medical compliance were collected from the study population through health records, questionnaires and health check-ups, as well as laboratory tests: fasting blood glucose, glycated haemoglobin, total cholesterol, triglycerides, low-density lipoprotein, high-density lipoprotein and field Measurement of the patient's systolic and diastolic blood pressure.

### Measurements and definitions

(1) smoking: people who were still smoking (not quit) at the time of the survey, whether or not they smoked daily [16]; (2) Alcohol consumption: defined as having consumed alcohol in the past 12 months [17]; (3) Physical activity: Adequate physical activity is defined as 150–300 min of moderate-intensity, or 75–150 min of vigorous-intensity physical activity, or some equivalent combination of moderate-intensity and vigorous-intensity aerobic physical activity per week [18] and insufficient physical activity is defined as physical activity but not meeting the criteria for adequate exercise. No physical activity is defined as not engaging in physical activity;(4) Hypertension: persons whose blood pressure measurement at the current year's health check-up is ≥ 140 mmHg systolic (1 mmHg = 0.1333 kPa) and/or 90 mmHg diastolic, or who have been diagnosed as hypertensive by a township (community) level hospital or above [19];(5) Dyslipidemia: those with triglycerides ≥ 2.26 mmol/L and/or total cholesterol ≥ 6.22 mmol/L and/or HDL cholesterol < 1.04 mmol/L and/or LDL cholesterol ≥ 4.14 mmol/L [20]; (6) body mass index (BMI) = Weight/height2 (kg/m2), according to the recommendations of the Chinese Guidelines for the Prevention and Control of Overweight and Obesity in Adults, BMI < 18.5 kg/m^2^ is considered low weight, 18.5 kg/m^2^ ≤ BMI ≤ 23.9 kg/m^2^ is normal weight, 24.0 kg/m^2^ ≤ BMI ≤ 27.9 kg/m^2^ is overweight, and BMI ≥ 28 kg/m^2^ was considered obesity [21]; (7) Glycemic control rate: The proportion of diabetic patients identified in this survey whose fasting blood glucose is currently controlled at < 7.0 mmol/L or whose glycated haemoglobin is < 7% [22].

### Statistical analysis

Statistical analysis of the data was performed using R4.1.2 software. Count data were expressed as rates and/or composition ratios with chi-square tests for comparison between groups, while measurement data were expressed as mean ± standard deviation with t-tests for comparison between groups. Multi-factor unconditional logistic regression analysis was used to analyse the factors affecting the rate of glycaemic control, followed by subgroup analysis for the presence of hypertension as well as hyperlipidemia, and the included variables with corresponding assignments are shown in Additional file 1: Table S1. differences were considered statistically significant at *P* < 0.05.

## Results

### Baseline information for people with diabetes

The 127,423 diabetic patients in this study were (69.5 ± 9.8) years old, of whom (68.6 ± 10.2) were male and (70.0 ± 9.4) were female, with a BMI of (24.8 ± 5.9) kg/m^2^ and a duration of diabetes of (8.7 ± 5.7) years in the survey population. The study population was predominantly married, middle school and high school/junior high school populations. There were 57,695 cases (45.28%) in the group with standard glycaemic control and 69,728 cases (54.72%) in the group with substandard diabetes control in this study. The differences between those who achieved and those who did not achieve the standard rate of diabetes control were statistically significant (*P* < 0.05) in terms of age, gender, years of diabetes diagnosis, family history of diabetes, and poor lifestyle and dietary behaviours. The difference in the history of hypertension and hyperlipidemia complications among the different glycemic controls was statistically significant (P < 0.05). In this study, the glycemic control rate in diabetic patients without hypertension and hyperlipidemia complications was 47.67%, compared with 52.54% and 37.24% in diabetic patients with hypertension alone and hyperlipidemia alone, respectively, and 41.80% in diabetic patients with both hypertension and hyperlipidemia (Table 1).

**Table 1:** Basic information on hypertension and dyslipidemia in type 2 diabetic patients in Guangzhou [n (%)]

Variable category	Male(n = 51,406)		Female(n = 76,017)		Total(n = 127,423)	
	Number of people	Composition ratio %	Number of people	Composition ratio %	Number of people	Composition ratio %
No hypertension or hyperlipidemia	12,683	42.65	17,053	57.35	29,736	23.34
Combined hypertension alone	14,323	39.66	21,795	60.34	36,118	28.34
Combined hyperlipidemia alone	10,516	40.20	15,641	59.80	26,157	20.53
Combined hypertension, hyperlipidemia	13,884	39.21	21,528	60.79	35,412	27.79
t/*X*^*2*^	92.151 < 0.001					
*P*						

### Co-morbidity of hypertension and hyperlipidemia in diabetic patients

In this study, 28.34% of diabetic patients had hypertension alone, 20.53% had hyperlipidemia alone, and 27.79% had complications of hypertension and hyperlipidemia compared to 23.34% of patients without hypertension and hyperlipidemia (Table 2).

**Table 2:** A multifactorial analysis of glycemic control in patients with diabetes mellitus combined with triple high co-morbidities in Guangzhou

	OR	OR 95% CI	*P* value
Model 1			
No hypertension or hyperlipidemia	Reference		
Combined hypertension alone	0.823	(0.798,0.849)	< 0.001
Combined hyperlipidemia alone	1.535	(1.484,1.588)	< 0.001
Combined hypertension, hyperlipidemia	1.268	(1.230,1.308)	< 0.001
Model 2			
No hypertension or hyperlipidemia	Reference		
Combined hypertension alone	0.834	(0.809,0.861)	< 0.001
Combined hyperlipidemia alone	1.534	(1.483,1.587)	< 0.001
Combined hypertension, hyperlipidemia	1.285	(1.246,1.326)	< 0.001
Model 3			
No hypertension or hyperlipidemia	Reference		
Combined hypertension alone	0.817	(0.791,0.843)	< 0.001
Combined hyperlipidemia alone	1.521	(1.470,1.574)	< 0.001
Combined hypertension, hyperlipidemia	1.25	(1.211,1.291)	< 0.001
OR = Odds ratio; CI = confidence intervals;			
Model 1: Unadjusted			
Model 2: Adjusted for age, gender,Education level,Marital status			
Model 3: Adjusted for Model 2 + years since diagnosis of diabetes, BMI, physical activity, smoking status, alcohol consumption, family history of diabetes,medical compliance			

### Glycemic control in T2DM patients combined with hypertension and hyperlipidemia

By adjusting for age, gender, education level, marital status, years since diagnosis of diabetes, BMI, physical activity, smoking status, alcohol consumption, family history of diabetes and medical compliance, glycemic control was better in diabetic patients with combined hypertension alone compared to those with uncomplicated diabetes (OR 0.817, 95% CI 0.791-0.843, *P* < 0.001), while those with combined hyperlipidemia alone (OR 1.521, 95% CI 1.470-1.574, *P* < 0.001) and diabetic patients with combined hypertension and hyperlipidaemia (OR 1.250, 95% CI 1.211-1.291, *P* < 0.001) had poor glycaemic control (Table 3).

**Table 3:** Multivariate unconditional logistic regression analysis of factors influencing diabetes control in type 2 diabetic patients in Guangzhou

Variable category		References	β	Sx^1^	Wald value	P value	OR	OR 95% CI
Gender	Female	Male	− 0.016	0.013	1.425	0.233	0.984	(0.959,1.010)
Age	≥ 60	< 60	− 0.108	0.016	45.779	< 0.001	0.897	(0.870,0.926)
Education level	High School/Secondary	Lower Secondary and below	− 0.067	0.015	18.802	< 0.001	0.936	(0.908,0.964)
	College and above		− 0.141	0.013	114.198	< 0.001	0.869	(0.847,0.891)
BMI(kg/m^2^)	< 18.5	18.5–24	− 0.161	0.039	17.355	< 0.001	0.851	(0.789,0.918)
	24–28		0.099	0.013	61.967	< 0.001	1.104	(1.077,1.131)
	> 28		0.130	0.017	60.514	< 0.001	1.139	(1.102,1.177)
Smoking	Yes	No	0.135	0.020	45.519	< 0.001	1.145	(1.101,1.190)
Drinking alcohol	yes	No	0.164	0.021	63.263	< 0.001	1.178	(1.131,1.226)
Physical activity	Not enough	No exercise	− 0.005	0.018	0.088	0.766	0.995	(0.959,1.031)
	Adequate		0.032	0.014	5.172	0.023	1.032	(1.004,1.061)
Medical compliance	fair	Good	0.053	0.013	17.111	< 0.001	1.054	(1.028,1.081)
	Poor		0.219	0.044	24.343	< 0.001	1.245	(1.141,1.358)
Family history of diabetes	Yes	No	0.150	0.024	39.233	< 0.001	1.161	(1.108,1.217)
Years since diagnosis of diabetes (years)	≥ 6	< 6	0.352	0.012	859.952	< 0.001	1.421	(1.388,1.455)

### Factors influencing glycemic control in patients with diabetes mellitus

The multivariate unconditional logistic regression analysis was conducted, with the dependent variable being whether the control status of the diabetic patient was up to standard and 12 factors as independent variables. Results showed that those aged ≥ 60 years (OR 0.897, 95% CI 0.870-0.926, *P* < 0.001), those with lower BMI (OR 0.851, 95% CI 0.789-0.918, *P* < 0.001), those with high school/middle school education and above (OR 0.936, 95% CI 0.908-0.964, *P* < 0.001 and OR 0.869, 95% CI 0.847-0.891, *P* < 0.001) had higher rates of diabetes control. Smoking (OR 1.145, 95% CI 1.101-1.190, *P* < 0.001), alcohol consumption (OR 1.178, 95% CI 1.131-1.226, *P* < 0.001), appropriate exercise (OR 1.032, 95% CI 1.004-1.061, *P* = 0.023), and diabetes diagnosis ≥ 6 years (OR 1.421, 95% CI 1.388-1.455, *P* < 0.001), fair or poor adherence (OR 1.054, 95% CI 1.028-1.081, *P* < 0.001 and OR 1.245, 95% CI 1.141-1.358, *P* < 0.001) and a family history of diabetes (OR 1.161, 95% CI 1.108-1.217, *P* < 0.001) had lower rates of diabetes control in patients with diabetes (Table 4).

**Table 4:** Univariate analysis of glycemic control in diabetic patients in Guangzhou

Variable category	Satisfactory control	Unsatisfactory control	t/*X*^*2*^	*P*
All	57,695(45.28%)	69,728(54.72%)		
age(SD),year	69.8(9.9)	69.1(9.7)	12.867	< 0.001
BMI(SD),kg/m2	24.6(5.0)	24.8(6.5)	− 6.594	< 0.001
Years since diagnosis of diabetes,year	8.2(5.4)	9.2(6.0)	− 33.276	< 0.001
Sex			54.628	< 0.001
Male	22,631(44.02%)	28,775(55.98%)		
Female	35,064(46.13%)	40,953(53.87%)		
Education level			110.770	< 0.001
Lower Secondary and below	26,796(43.94%)	34,189(56.06%)		
High School/Secondary	11,635(45.27%)	14,068(54.73%)		
College and above	19,264(47.29%)	21,471(52.71%)		
Marital status			4.304	0.231
Unmarried	419(44.41%)	527(55.59%)		
Married	52,011(45.20%)	63,065(54.80%)		
Widowed	4833(46.20%)	5628(53.80%)		
Divorced	430(45.84%)	508(54.16%)		
Smoking			117.020	< 0.001
Yes	6301(41.19%)	8996(58.81%)		
No	51,394(45.84%)	60,732(54.16%)		
Drinking alcohol			153.990	< 0.001
Yes	5365(40.21%)	7976(59.79%)		
No	52,330(45.87%)	61,752(54.13%)		
Physical activity			13.021	0.001
No exercise	14,007(45.82%)	16,563(54.18%)		
Not enough	9185(46.01%)	10,778(53.99%)		
Adequate	34,503(44.87%)	42,387(55.13%)		
Family history of diabetes			43.117	< 0.001
Yes	3296(41.71%)	4606(58.29%)		
No	54,399(45.51%)	65,122(54.49%)		
Medical compliance			74.399	< 0.001
Good	40,105(46.00%)	47,081(54.00%)		
Fair	16,707(43.96%)	21,298(56.04%)		
Poor	883(39.56%)	1349(60.44%)		
Disease comorbidity status			1691.400	< 0.001
No hypertension or hyperlipidemia	14,176(47.67%)	15,560(52.33%)		
Combined hypertension alone	18,975(52.54%)	17,143(47.46%)		
Combined hyperlipidemia alone	9741(37.24%)	16,416(62.76%)		
Combined hypertension, hyperlipidemia	14,803(41.80%)	20,609(58.20%)		

### Subgroup analysis

**Fig. 1:**
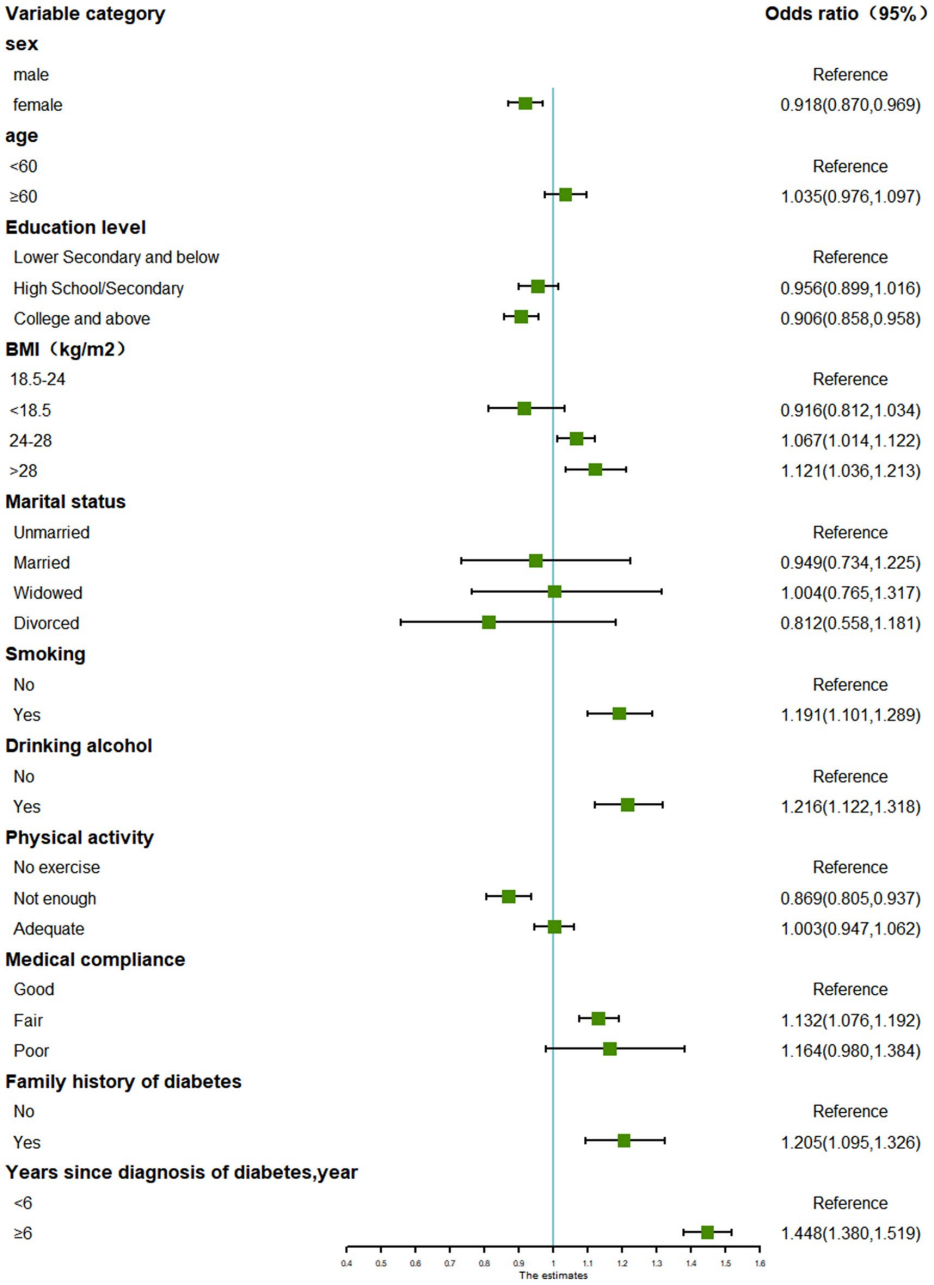
Forest plot for subgroup analysis of glycemic control in T2D patients without hypertension and hyperlipidemia

**Fig. 2:**
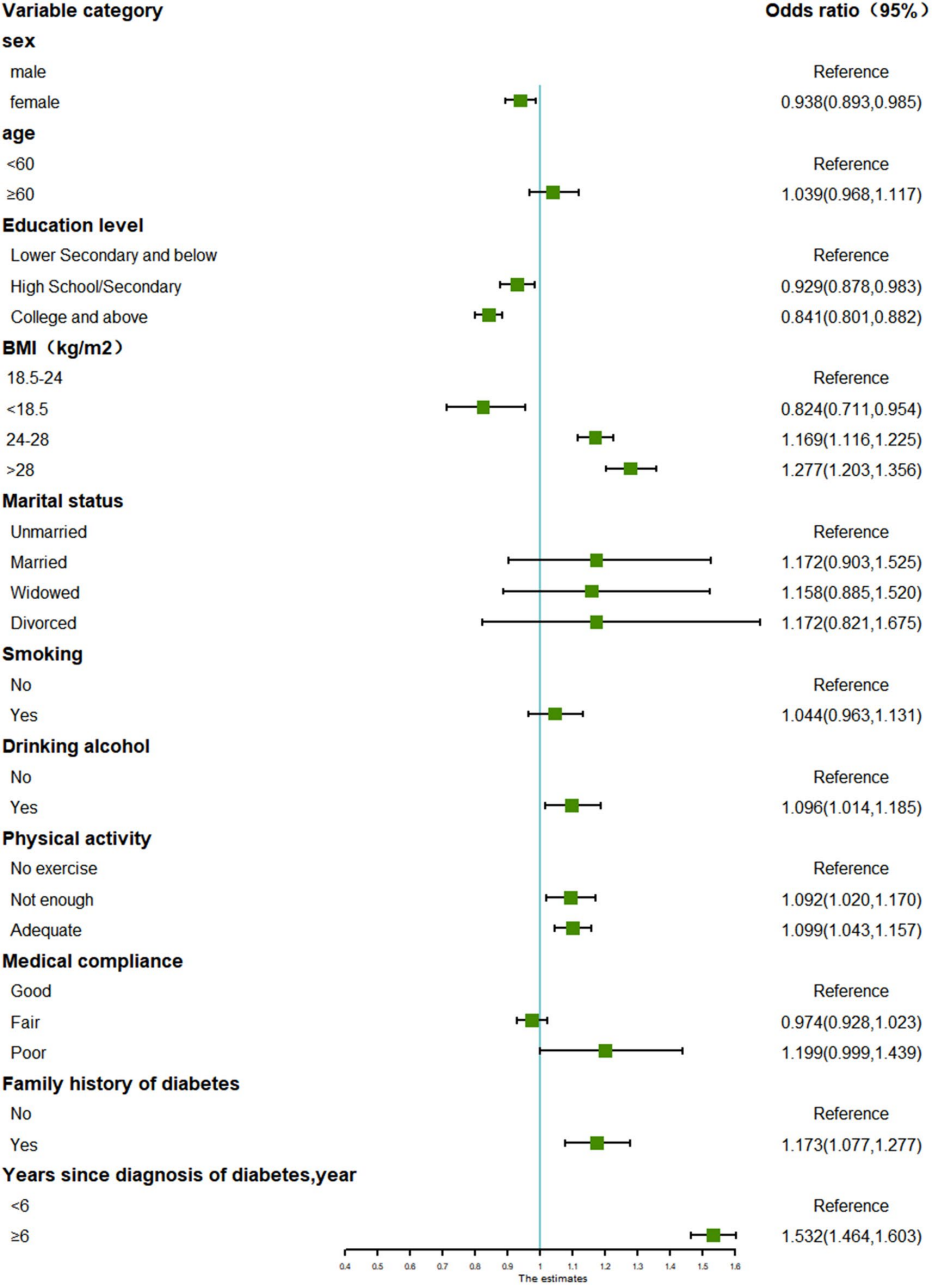
Forest plot for subgroup analysis of glycemic control in patients with T2D in combination with hypertension

**Fig. 3:**
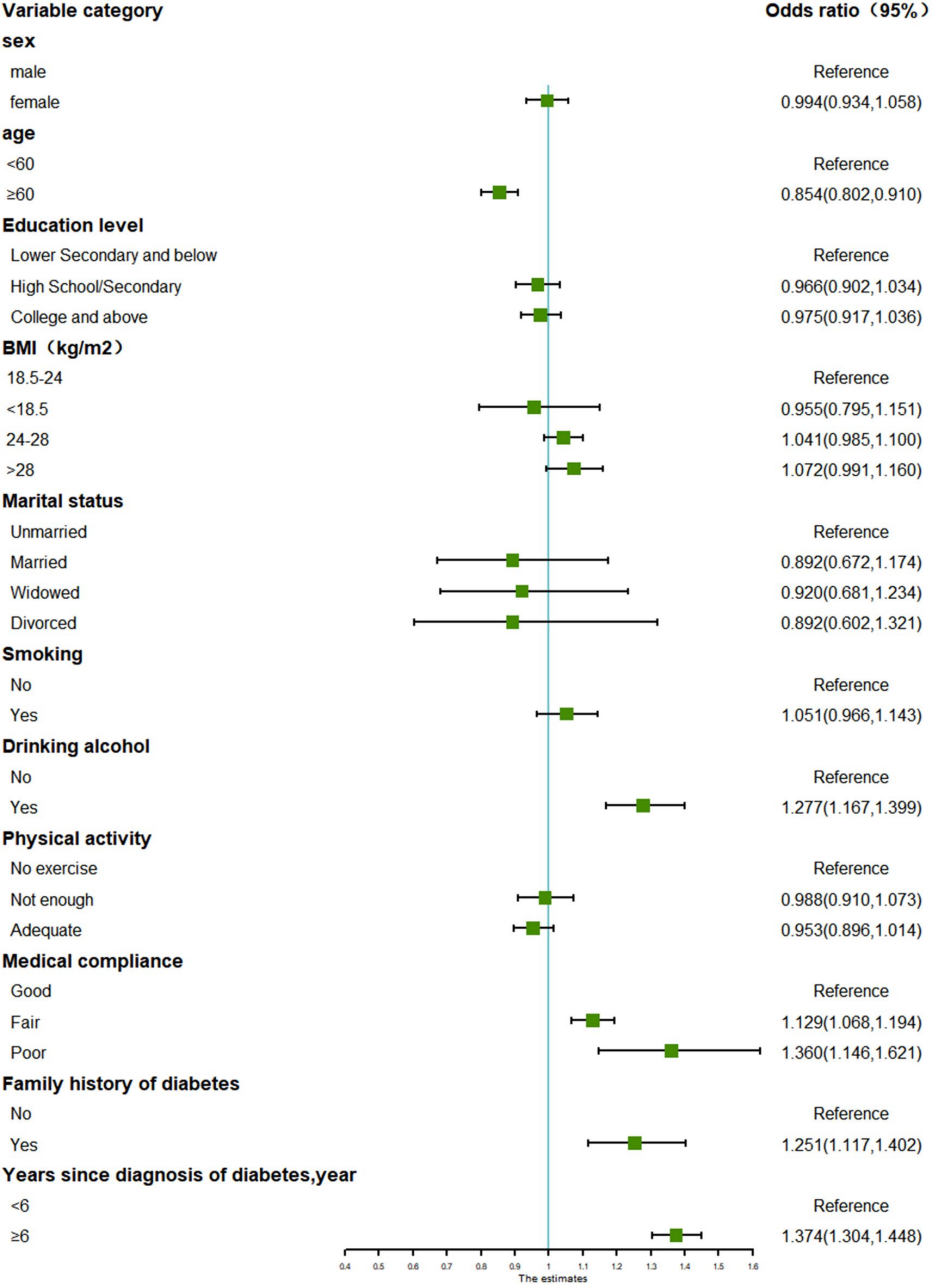
Forest plot for subgroup analysis of glycemic control in T2D patients with comorbid hyperlipidemia

**Fig. 4:**
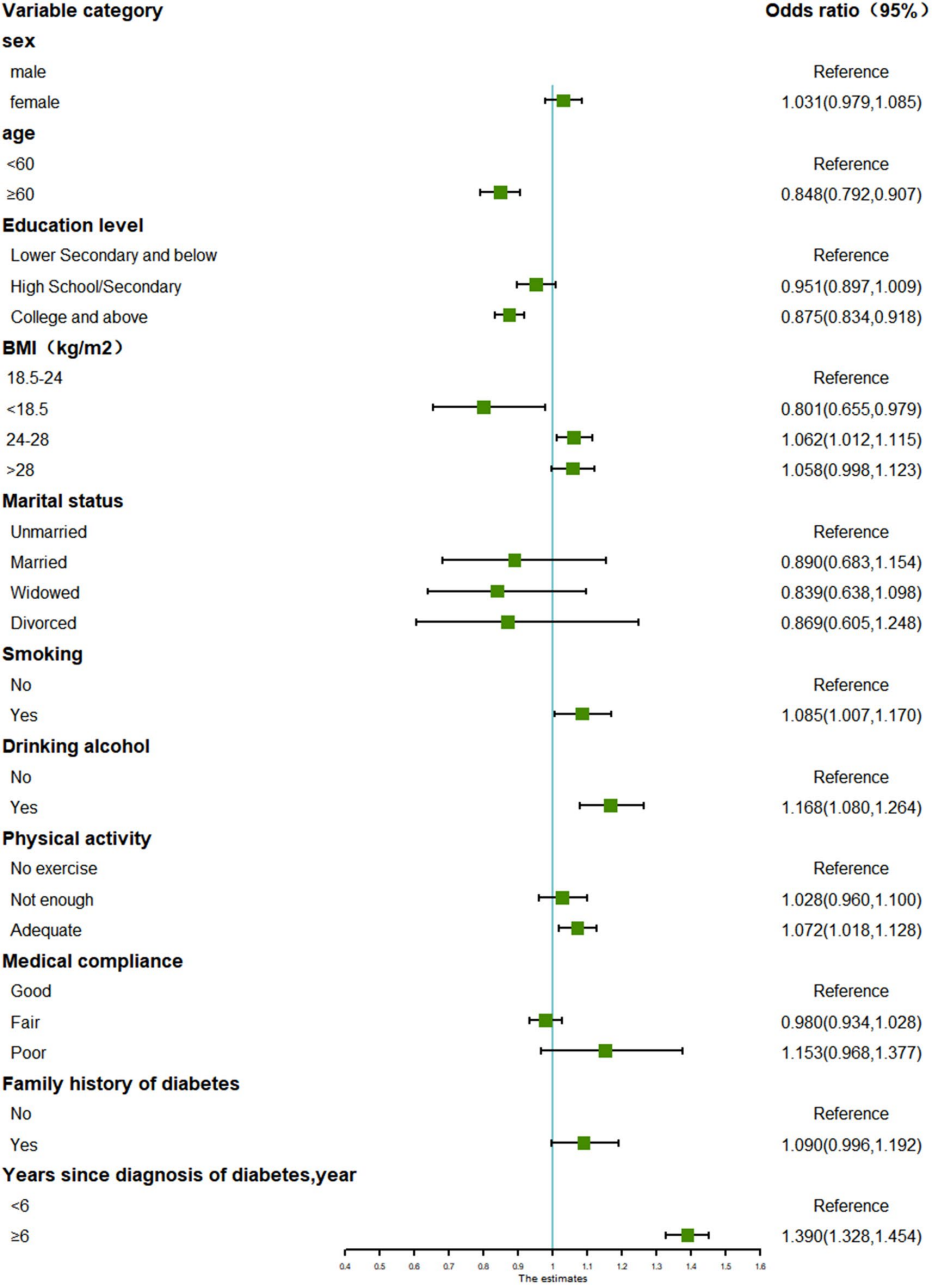
Forest plot for subgroup analysis of glycemic control in T2D patients with combined hypertension and hyperlipidemia

This study also analysed subgroups of people with and without complications of hypertension and hyperlipidaemia. The results showed that overweight and obesity, smoking, alcohol consumption, poor medical adherence, family history of diabetes and long years of diabetes diagnosis were risk factors for glycaemic control in all diabetic patients, regardless of the presence or absence of hypertension and hyperlipidaemic complications. (Figs. 1, 2, 3, 4).

## Discussion

This study assessed the glycemic control rate and the factors affecting the glycemic control rate in patients with type 2 diabetes combined with high blood lipids and hypertension in Guangzhou in 2020. The results showed that the overall glycemic control rate of diabetic patients in Guangzhou in 2020 was 45.28%, previous studies investigating glycaemic control rates in the Chinese diabetic population have ranged from 49.2% to 68.5% [23–27]. This suggests that the overall glycaemic control situation of diabetic patients in Guangzhou is critical. All we need to improve the glycaemic control rate of diabetic patients by addressing the weaknesses in diabetes management.

The results of this study showed that diabetic patients with combined hyperlipidaemia had a lower control rate compared to diabetic patients without any complications (37.24% vs. 47.67%) because free fatty acids, as precursors to hepatic glucose metabolism, increase apoptosis of pancreatic β-cells and increase muscle insulin resistance[28], leading to impaired insulin secretion and persistent hyperglycaemia [29], which requires diabetic patients to be aware of abnormalities in their lipid metabolism while controlling their blood glucose.

Previous studies have shown that hypertension is an independent risk factor for diabetes, and that hypertension increases C-reactive protein, interleukins and inflammatory markers such as adhesion molecules related to insulin signalling pathways and β-cell function, and further promotes the development of diabetes [30, 31]. In this study, it had shown the higher control rate of diabetic patients with combined hypertension.The reason may be due to the integration of diabetes and hypertension into community management, which allowed people with combined hypertension and diabetes to receive better basic public health services in the community.

In this study, we analysed the factors influencing glycaemic control in patients with diabetes as well as in patients with diabetic comorbidities, and the results showed that different factors have different effects on glycaemic control. Diabetes control rates were lower in men with T2DM than in women, which may be due to differences in hormone levels, body fat distribution and adherence between men and women [23]. There is a trend for diabetes control rates to increase with age, possibly because older people have more time and energy to focus on their health, and consultation time and opportunities increase. This study also observed a reduced risk of hyperglycaemia in those with higher levels of education, possibly due to their better understanding of diabetes, obesity risk and glycaemic control [32]. The lower rate of diabetes control in patients with a longer duration of diabetes may be due to a decrease in insulin secretion over time as the patient's own islet cells fail with the progression of diabetes [33]. Studies have shown that family history increases the risk of diabetes mainly through lifestyle clustering, genetic entrenchment and genetic susceptibility [34], which is consistent with the results of this study. The results of this study also showed that overweight and obese individuals have lower rates of diabetes control due to the fact that body fat accumulation causes reduced glucose tolerance and thus reduced use of glucose by tissues such as muscle [35]. In addition, smoking and alcohol consumption reduce diabetes control rates because nicotine exposure may induce a pro-inflammatory metabolic state, which affects insulin sensitivity and β-cell function [36]. Previous epidemiological studies have suggested that alcohol consumption leads to an increased risk of diabetes [37], but the exact mechanisms need further study. Moderate physical activity can improve blood glucose levels [38], and in our study, physical activity was a risk factor for diabetes control rate, which is inconsistent with the results of previous studies. The reason may be that there was information bias or confounding bias in this study when collecting data on physical activity in diabetic patients, and the content of the questionnaire and survey method should be improved in the future to obtain more accurate data.

## Limitations and strengths

As a strength of this study, the relatively large sample size in this study allows for a more real world study of the accuracy of the data and is more convincing. Fasting blood glucose and glycosylated haemoglobin were also used in this study to represent blood glucose levels to show the true level of glycaemic control. In addition, some limitations of this study should be noted. Firstly, some subjects were excluded because they did not fit the purpose of the study and/or their blood glucose or other data were incomplete, so there may have been selection bias. Then the data for this study were obtained through a cross-sectional survey. Further studies are therefore needed to explore associations in the longitudinal setting. Nonetheless, we provide a comprehensive picture of the current profile and glycaemic control rates of patients with triple hypertension in Guangzhou, and identify risk factors for disease management and other aspects that influence glycaemic control.

## Conclusion

In conclusion, the prevalence of hypertension, hyperlipidaemia and glycaemic control in type 2 diabetic patients in Guangzhou is not encouraging.The public health program is a health guidance for diabetic lifestyle and lifestyle interventions to control blood glucose, followed by regular blood glucose monitoring and screening for complications. The implementation of the public health program helps diabetic patients to understand about the disease, while controlling blood glucose levels, reducing the occurrence and development of diabetes and complications, and ultimately improving the quality of life, so it is important to further strengthen the standardised management of diabetic patients, pay attention to the follow-up management of diabetic patients, especially those with hyperlipidaemia.It was very necessary to bring dyslipidemia patients into the management of chronic disease patients in the community, so as to realize the co-management of hypertension, hyperglycemia and hyperlipidemia. Therefore, it is very important for the T2DM patients to maintain healthy lifestyle and dietary habits, and control their weight in order to lower blood glucose levels, and reduce the incidence and prevalence of diabetic complications.

## Supplementary Information

**Additional file 1: Table S1.** Logistic regression variable assignment. **Table S2.** A subgroup analysis of complications in a multifactorial unconditional logistic regression analysis of factors influencing glycemic control in type 2 diabetic patients in Guangzhou. **Table S3.** Univariate analysis of complications in diabetic patients in Guangzhou.

## Data Availability

The datasets analysed during the current study are available from the corresponding author upon reasonable request.
