# Supplementary material for: The hypertension and hyperlipidemia status among type 2 diabetic patients in the community and influencing factors analysis of glycemic control
Source: Diabetol Metab Syndr. 2023 Apr 13;15:73. doi: 10.1186/s13098-023-01013-0 (PMC10100166; doi:10.1186/s13098-023-01013-0)
Supplement: Supplementary file 1 — Additional file 1: Table S1. Logistic regression variable assignment. Table S2. A subgroup analysis of complications in a multifactorial unconditional logistic regression analysis of factors influencing glycemic control in type 2 diabetic patients in Guangzhou. Table S3. Univariate analysis of complications in diabetic patients in Guangzhou. [file 13098_2023_1013_MOESM1_ESM.docx]

| Table S1 Logistic regression variable assignment | |
| --- | --- |
| Variable category | Variable assignment |
| Blood glucose control | 1= Not met 0= Met |
| Age, years | 1= <60 2= ≥60 |
| Gender | 1=Male 2=Female |
| Education level | 1=Junior high school and below 2=High school/junior college 3=College and above |
| Marital status | 1= unmarried 2= married 3= widowed 4= divorced |
| BMI, kg/m2 | 1=18.5-24 2=<18.5 3=24-28 4=>28 |
| Duration of diabetes, years | 1= <6 2= ≥6 |
| Current Smoking | 1=Yes 0=No |
| Current alcohol consumption | 1= Yes 0= No |
| Physical activity | 0= No campaign 1= Insufficient 2= Sufficient |
| Family history of diabetes | 1=Yes 0=No |
| Medical compliance behaviour | 1=good 2=fair 3=poor |
| History of disease | 1=no hypertension, hyperlipidemia 2=combined hypertension alone 3=combined hyperlipidemia alone 4=combined hypertension, hyperlipidemia |

| Table S2 A subgroup analysis of complications in a multifactorial unconditional logistic regression analysis of factors influencing glycemic control in type 2 diabetic patients in Guangzhou | | | | | | | | |
| --- | --- | --- | --- | --- | --- | --- | --- | --- |
| No hypertension or hyperlipidemia（n=29736） | | | | | | | | |
| Variable category |  | Reference | β | Sx¹ | Wald value | P value | OR | OR 95 % CI |
| Gender | Female | Male | -0.085 | 0.028 | 9.562 | 0.002 | 0.918 | (0.870,0.969) |
| Age | ≥60 | <60 | 0.034 | 0.030 | 1.335 | 0.248 | 1.035 | (0.976,1.097) |
| Education level | High School/Secondary | Lower Secondary and below | -0.045 | 0.031 | 2.092 | 0.148 | 0.956 | (0.899,1.016) |
|  | College and above |  | -0.098 | 0.028 | 12.233 | < 0.001 | 0.906 | (0.858,0.958) |
| BMI（kg/m2） | <18.5 | 18.5-24 | -0.087 | 0.062 | 2.000 | 0.157 | 0.916 | (0.812,1.034) |
|  | 24-28 |  | 0.065 | 0.026 | 6.264 | 0.012 | 1.067 | (1.014,1.122) |
|  | >28 |  | 0.114 | 0.040 | 8.008 | 0.005 | 1.121 | (1.036,1.213) |
| Marital status | Married | Unmarried | -0.053 | 0.130 | 0.164 | 0.685 | 0.949 | (0.734,1.225) |
|  | Widowed |  | 0.004 | 0.138 | 0.001 | 0.975 | 1.004 | (0.765,1.317) |
|  | Divorced |  | -0.209 | 0.191 | 1.191 | 0.275 | 0.812 | (0.558,1.181) |
| Smoking | Yes | No | 0.175 | 0.040 | 18.818 | < 0.001 | 1.191 | (1.101,1.289) |
| Drinking alcohol | yes | No | 0.195 | 0.041 | 22.727 | < 0.001 | 1.216 | (1.122,1.318) |
| Physical activity | Not enough | No exercise | -0.141 | 0.039 | 13.394 | < 0.001 | 0.869 | (0.805,0.937) |
|  | Adequate |  | 0.003 | 0.029 | 0.010 | 0.920 | 1.003 | (0.947,1.062) |
| Medical compliance | fair | Good | 0.124 | 0.026 | 22.588 | < 0.001 | 1.132 | (1.076,1.192) |
|  | Poor |  | 0.152 | 0.088 | 2.994 | 0.084 | 1.164 | (0.980,1.384) |
| Family history of diabetes | Yes | No | 0.186 | 0.049 | 14.657 | < 0.001 | 1.205 | (1.095,1.326) |
| Years since diagnosis of diabetes (years) | ≥6 | <6 | 0.370 | 0.025 | 226.346 | < 0.001 | 1.448 | (1.380,1.519) |
| Combined hypertension alone（n=36118） | | | | | | | | |
| Variable category |  | Reference | β | Sx¹ | Wald value | P value | OR | OR 95 % CI |
| Gender | Female | Male | -0.064 | 0.025 | 6.697 | 0.010 | 0.938 | (0.893,0.985) |
| Age | ≥60 | <60 | 0.039 | 0.037 | 1.114 | 0.291 | 1.039 | (0.968,1.117) |
| Education level | High School/Secondary | Lower Secondary and below | -0.074 | 0.029 | 6.602 | 0.010 | 0.929 | (0.878,0.983) |
|  | College and above |  | -0.174 | 0.025 | 50.116 | < 0.001 | 0.841 | (0.801,0.882) |
| BMI（kg/m2） | <18.5 | 18.5-24 | -0.193 | 0.075 | 6.664 | 0.010 | 0.824 | (0.711,0.954) |
|  | 24-28 |  | 0.157 | 0.024 | 43.526 | < 0.001 | 1.169 | (1.116,1.225) |
|  | >28 |  | 0.245 | 0.031 | 63.538 | < 0.001 | 1.277 | (1.203,1.356) |
| Marital status | Married | Unmarried | 0.158 | 0.134 | 1.402 | 0.236 | 1.172 | (0.903,1.525) |
|  | Widowed |  | 0.147 | 0.138 | 1.139 | 0.286 | 1.158 | (0.885,1.520) |
|  | Divorced |  | 0.159 | 0.182 | 0.760 | 0.383 | 1.172 | (0.821,1.675) |
| Smoking | Yes | No | 0.043 | 0.041 | 1.101 | 0.294 | 1.044 | (0.963,1.131) |
| Drinking alcohol | yes | No | 0.092 | 0.040 | 5.345 | 0.021 | 1.096 | (1.014,1.185) |
| Physical activity | Not enough | No exercise | 0.088 | 0.035 | 6.296 | 0.012 | 1.092 | (1.020,1.170) |
|  | Adequate |  | 0.094 | 0.026 | 12.637 | < 0.001 | 1.099 | (1.043,1.157) |
| Medical compliance | fair | Good | -0.026 | 0.025 | 1.103 | 0.294 | 0.974 | (0.928,1.023) |
|  | Poor |  | 0.182 | 0.093 | 3.804 | 0.051 | 1.199 | (0.999,1.439) |
| Family history of diabetes | Yes | No | 0.159 | 0.044 | 13.365 | < 0.001 | 1.173 | (1.077,1.277) |
| Years since diagnosis of diabetes (years) | ≥6 | <6 | 0.426 | 0.023 | 341.368 | < 0.001 | 1.532 | (1.464,1.603) |
| Combined hyperlipidemia alone（n=26157） | | | | | | | | |
| Variable category |  | Reference | β | Sx¹ | Wald value | P value | OR | OR 95 % CI |
| Gender | Female | Male | -0.006 | 0.032 | 0.030 | 0.862 | 0.994 | (0.934,1.058) |
| Age | ≥60 | <60 | -0.158 | 0.032 | 23.544 | <0.001 | 0.854 | (0.802,0.910) |
| Education level | High School/Secondary | Lower Secondary and below | -0.035 | 0.035 | 0.987 | 0.321 | 0.966 | (0.902,1.034) |
|  | College and above |  | -0.025 | 0.031 | 0.663 | 0.416 | 0.975 | (0.917,1.036) |
| BMI（kg/m2） | <18.5 | 18.5-24 | -0.046 | 0.094 | 0.235 | 0.628 | 0.955 | (0.795,1.151) |
|  | 24-28 |  | 0.040 | 0.028 | 2.036 | 0.154 | 1.041 | (0.985,1.100) |
|  | >28 |  | 0.069 | 0.040 | 2.983 | 0.084 | 1.072 | (0.991,1.160) |
| Marital status | Married | Unmarried | -0.114 | 0.142 | 0.648 | 0.421 | 0.892 | (0.672,1.174) |
|  | Widowed |  | -0.083 | 0.151 | 0.304 | 0.582 | 0.920 | (0.681,1.234) |
|  | Divorced |  | -0.114 | 0.200 | 0.325 | 0.569 | 0.892 | (0.602,1.321) |
| Smoking | Yes | No | 0.049 | 0.043 | 1.320 | 0.251 | 1.051 | (0.966,1.143) |
| Drinking alcohol | yes | No | 0.245 | 0.046 | 28.119 | <0.001 | 1.277 | (1.167,1.399) |
| Physical activity | Not enough | No exercise | -0.012 | 0.042 | 0.081 | 0.776 | 0.988 | (0.910,1.073) |
|  | Adequate |  | -0.048 | 0.031 | 2.340 | 0.126 | 0.953 | (0.896,1.014) |
| Medical compliance | fair | Good | 0.122 | 0.028 | 18.377 | <0.001 | 1.129 | (1.068,1.194) |
|  | Poor |  | 0.308 | 0.088 | 12.134 | <0.001 | 1.360 | (1.146,1.621) |
| Family history of diabetes | Yes | No | 0.224 | 0.058 | 14.874 | <0.001 | 1.251 | (1.117,1.402) |
| Years since diagnosis of diabetes (years) | ≥6 | <6 | 0.318 | 0.027 | 142.071 | <0.001 | 1.374 | (1.304,1.448) |
| Combined hypertension, hyperlipidemia（n=35412） | | | | | | | | |
| Variable category |  | Reference | β | Sx¹ | Wald value | P value | OR | OR 95 % CI |
| Gender | Female | Male | 0.030 | 0.026 | 1.347 | 0.246 | 1.031 | (0.979,1.085) |
| Age | ≥60 | <60 | -0.165 | 0.035 | 22.842 | < 0.001 | 0.848 | (0.792,0.907) |
| Education level | High School/Secondary | Lower Secondary and below | -0.050 | 0.030 | 2.775 | 0.096 | 0.951 | (0.897,1.009) |
|  | College and above |  | -0.134 | 0.025 | 29.272 | < 0.001 | 0.875 | (0.834,0.918) |
| BMI（kg/m2） | <18.5 | 18.5-24 | -0.222 | 0.102 | 4.718 | 0.030 | 0.801 | (0.655,0.979) |
|  | 24-28 |  | 0.060 | 0.025 | 5.890 | 0.015 | 1.062 | (1.012,1.115) |
|  | >28 |  | 0.057 | 0.030 | 3.528 | 0.060 | 1.058 | (0.998,1.123) |
| Marital status | Married | Unmarried | -0.116 | 0.134 | 0.758 | 0.384 | 0.890 | (0.683,1.154) |
|  | Widowed |  | -0.175 | 0.138 | 1.609 | 0.205 | 0.839 | (0.638,1.098) |
|  | Divorced |  | -0.140 | 0.185 | 0.578 | 0.447 | 0.869 | (0.605,1.248) |
| Smoking | Yes | No | 0.082 | 0.038 | 4.555 | 0.033 | 1.085 | (1.007,1.17) |
| Drinking alcohol | yes | No | 0.155 | 0.040 | 15.007 | < 0.001 | 1.168 | (1.080,1.264) |
| Physical activity | Not enough | No exercise | 0.028 | 0.035 | 0.634 | 0.426 | 1.028 | (0.960,1.100) |
|  | Adequate |  | 0.069 | 0.026 | 6.978 | 0.008 | 1.072 | (1.018,1.128) |
| Medical compliance | fair | Good | -0.021 | 0.025 | 0.704 | 0.401 | 0.980 | (0.934,1.028) |
|  | Poor |  | 0.143 | 0.090 | 2.527 | 0.112 | 1.153 | (0.968,1.377) |
| Family history of diabetes | Yes | No | 0.086 | 0.046 | 3.514 | 0.061 | 1.090 | (0.996,1.192) |
| Years since diagnosis of diabetes (years) | ≥6 | <6 | 0.329 | 0.023 | 203.723 | < 0.001 | 1.390 | (1.328,1.454) |

| Table S3 Univariate analysis of complications in diabetic patients in Guangzhou | | | | | | |
| --- | --- | --- | --- | --- | --- | --- |
| Variable category | No hypertension or hyperlipidemia | Combined hypertension alone | Combined hyperlipidemia alone | Combined hypertension, hyperlipidemia | F/X2 | *P* |
| All | 29736(23.34%) | 36118(28.34%) | 26157(20.53%) | 35412(27.79%) |  |  |
| age(SD),year | 67.4(10.0) | 71.8(9.3) | 66.9(9.9) | 70.6(9.3) | 1913.000 | <0.001 |
| sex |  |  |  |  | 92.151 | <0.001 |
| male | 12683(24.67%) | 14323(27.86%) | 10516(20.46%) | 13884(27.01%) |  |  |
| female | 17053(22.43%) | 21795(28.67%) | 15641(20.58%) | 21528(28.32%) |  |  |
| Education level |  |  |  |  | 1016.700 | <0.001 |
| Lower Secondary and below | 15028(24.64%) | 15799(25.91%) | 14098(23.12%) | 16060(26.33%) |  |  |
| High School/Secondary | 6156(23.95%) | 7627(29.67%) | 5070(19.73%) | 6850(26.65%) |  |  |
| College and above | 8552(20.99%) | 12692(31.16%) | 6989(17.16%) | 12502(30.69%) |  |  |
| Marital status |  |  |  |  | 395.840 | <0.001 |
| Unmarried | 243(25.63%) | 234(24.68%) | 231(24.37%) | 240(25.32%) |  |  |
| Married | 27283(23.71%) | 32026(27.83%) | 24048(20.9%) | 31719(27.56%) |  |  |
| Widowed | 2004(19.16%) | 3593(34.35%) | 1664(15.91%) | 3200(30.59%) |  |  |
| Divorced | 206(21.96%) | 265(28.25%) | 214(22.81%) | 253(26.97%) |  |  |
